# Correlates of cigarette smoking among school-going adolescents in Thailand: findings from the Thai global youth tobacco survey 2005

**DOI:** 10.1186/1755-7682-1-8

**Published:** 2008-06-11

**Authors:** Emmanuel Rudatsikira, Adamson S Muula, Seter Siziya, Ronald H Mataya

**Affiliations:** 1 Departments of Epidemiology and Biostatistics, School of Public Health, Loma Linda University, Loma Linda, California, USA; 2 Department of Community Medicine and Public Health, College of Medicine, University of Malawi, Blantyre, Malawi; 3 Department of Community Medicine, University of Zambia Medical School, Lusaka, Zambia; 4 Department of Global Health, School of Public Health, Loma Linda University, California, USA

## Abstract

**Background:**

Many studies examining the social correlates of tobacco use among adolescents fail to recognise theories of health behaviour and health promotion in their analysis. Using the Socio-Ecologiocal Model (SEM) we assessed the demographic and social factors associated with current cigarette smoking among adolescents in Thailand.

**Method:**

A secondary analysis of cross-sectional data from the Thai Global Youth Tobacco Survey (Thai GYTS) 2005 was analysed to obtain prevalence of selected attributes and assess factors associated with current cigarette smoking. Current cigarette smoking was defined as having smoked a cigarette, even a single puff, in the last 30 days. Logistic regression was conducted to estimate the level of association between the explanatory variables and current smoking.

**Results:**

Of the 18,368 respondents, 22.0% males and 5.2% females reported being current smokers (p < 0.001). In multivariate logistic regression analysis, current smoking was negatively associated with the perception that smoking is harmful to health (OR = 0.47; 95% CI [0.33, 0.66]) and positively associated with male gender (OR = 3.46; 95% CI [2.72, 4.86]) and having smoking parents (OR = 1.62; 95% CI [1.25, 2.11]) and friends (OR = 5.07; 95% CI [3.54, 7.25]) for some friends smokers and OR = 26.71; 95% CI [18.26, 39.06] for most or all friends smokers. Compared to subjects 11 = 12 year olds, those who were older were less likely to report smoking (OR = 0.49; 95% CI [0.36, 0.66] for 13 years olds, OR = 0.56; 95% CI [0.40, 0.79] for 14 years olds, OR = 0.59; 95% CI [0.41, 0.86] for 15 years olds).

**Conclusion:**

Current cigarette smoking was associated with male gender, smoking parents or closest peers. Perception that smoking was harmful to health was associated with less likelihood of being a current smoker.

## Introduction

Tobacco is the leading cause of morbidity and mortality globally [1-3]. In Thailand current adult daily and occasional tobacco smoking prevalence was reported at 37.2% for males, 2.1% for females and19.5% overall [4]. Tobacco farming is an important economic activity in the country. In 1995, 0.21% of Thailand's land was used for tobacco farming [5]. In 1998, 0.181 billion cigarette sticks were exported while 1.7 billion sticks were imported by the country [5]. The Thai tobacco industry is largely dominated by the state-owned Thai Tobacco Monopoly (TTM). The tobacco industry contributed 3.5%-4.5% of the total government revenue in the country between 2000 and 2005 [6].

Although the country derives significant financial resources from tobacco, it has two specific laws regarding tobacco use control since 1992 i.e. the Tobacco Products Control Act of 1992 and Non-smokers' Health Protection Act of 1992; the Tobacco Products Control Act of 1992 includes total advertising ban [7,8].

Tobacco use is an important public health concern. Previous research has linked substance use, such as tobacco use, to other health-related risky behaviours, mental health problems, suicide, motor vehicle accidents, violent crime, dental problems and other major health problems, including cancer and heart disease [9-13]. Research on the sequence of drug use suggests that cigarettes and alcohol may serve as "gateway" drugs to illicit drugs [10]. As the adolescent and young adult years represent a critical period for the initiation of substance use this period also avails itself as critical in the institution of interventions to prevent substance use. Such public health interventions should, as far as is practicable, be based on an understanding of the many factors that shape use during the adolescent and young adult years.

Attempts to understand adolescent health behaviours have not always been linked to theories of health behaviours. We hypothesized that adolescents' cigarette use may be explainable through the Social ecological model (SEM) of health behaviour [14-17]. The SEM has been applied to a broad range of health behaviours and among different subject populations. The model suggests that various factors including individual-level factors and the socio-cultural environment that an individual is exposed to contribute or interact to produce specific behaviors such as cigarette smoking. With regard to adolescent smoking, we hypothesized that adolescents who perceived that smoking was harmful to their health would be less likely to be current smokers. Belief that smoking is harmful, which may be influenced by education or thorough observation within one's society, as well as an individual's age. One's sex (which may influence gender) and smoking among peers and parents may also be perceived as operating within the SEM. If society is more permissive toward smoking by one gender, such gender may be more likely to smoke. Furthermore, adolescents who have their peers or parents who also smoke may be living in environments that is more tolerant towards smoking. We also aimed to assess other socio-demographic characteristics that could be associated with being a current cigarette smoker.

## Methods

### Sampling design and study participant recruitment

The current study was based on secondary analysis of data from the 2005 Thai Global Youth Tobacco Survey. Comprehensive descriptions of the Global Youth Tobacco Survey (GYTS) purpose and methodology have been described in the literature elsewhere [18-20]. In brief, the GYTS uses a standard survey questionnaire with core questions and country-specific questions. The core questions are the same in all the countries where the survey is administered. The survey recruits in-school adolescents in classes with the majority of its students are between 13–15 years. A two-stage clustered sampling survey approach is used in which the primary sampling units are schools with students in the eligible age group (13 to 15 years). At the second stage of sampling, eligible classes (classes with the majority of students 13 to 15 years) within the selected schools are randomly selected. All students within the selected classes are eligible to participate in the study, regardless of their actual ages. However, students are free not to participate when identified as eligible for participation.

The GYTS data in this report include surveys completed in the following 5 regions in Thailand: Bangkok; Central-Western; North Eastern; Northern, and Southern. The school response rate was 100% while the student response rate was 99%.

Study participants self-complete questionnaires which had standard "core questions" and a few other country-specific questions [18-20]. Questionnaires which were administered and anonymously self-completed within class time. Time taken to complete questionnaire was estimated at about 40 minutes. Some of the questions asked aimed to collect information on the following variables: age, history of having ever smoked, current smoking status, parental smoking, having friends who smoked, perception that smoking is harmful to health, exposure to tobacco advertisements and approval of smoking among boys and girls. These explanatory variables have been previously reported as being associated with smoking among adolescents in other settings [21-25].

Completed questionnaires were scanned by computer and data file generated in Access.

### Data analysis

Data were analyzed by SUDAAN software 9.0 (Research Triangle Institute, Research Triangle Park, North Carolina, United States). We generated frequencies and 95% confidence intervals of relevant variables such as demographics, approval of smoking among boys and girls and exposure to tobacco advertisements. To assess the associations between a selected list of variables identified from the literature as possibly associated with current cigarette smoking, logistic regression analysis was conducted both at bivariate and multivariate level. These explanatory variables were: gender; age; peer smoking; and parental smoking. Based on the SEM, we hypothesised that adolescents who belief that smoking was harmful to one's health, age, sex, peer and parental smoking would be associated with being a current cigarette smoker. Current smoking was defined as having ever smoked, even a single puff in the past 30 days preceding the survey.

The bivariate logistic regression analysis was conducted to obtained unadjusted odds ratios with current smoking status (Yes or Not) as the outcome of interest and each of the following variables as explanatory or predictor variables (age, sex, peer smoking, parental smoking, perception that smoking is harmful). Separate multivariable models were also conducted with the same outcome variable but each of the explanatory variables as the main exposure of interest with the rest of the variables being controlled for.

## Results

### Characteristics of the study participants

A total of 18,368 students participated in the study of whom 9,851 (51.4%) were males and 9,312 (48.6%) were females. The median age was 14 years.

### Prevalence of current and ever cigarette smoking

Of the 18,368 participants, 22.0%; 95% CI [19.8, 24.4] males and 5.2%; 95% CI [4.3, 6.2] females reported being current smokers (p < 0.001). An estimated 42.9%; 95% CI [40.7, 45.3] males and 18.4%; 95% CI [15.9, 21.1] females had ever smoked a cigarette (p < 0.001).

### Prevalence to cigarette advertisements and attitudes towards smoking

Tables 1 and 2 show the prevalence of various exposures to pro-tobacco advertisements and perceptions towards smoking among the study sample. More than a third of the sample had seen tobacco advertisements on television in past 30 days, had an item with tobacco brand logo and had seen tobacco advertisements on the internet. Furthermore smokers were perceived favourably (see Table 1).

**Table 1:** Exposure to tobacco advertisement among adolescents in Thailand

Characteristic	Number of participants	% of total and 95% CI
Seen cigarette brand name on TV in past 30 days	13,037	33.8 (30.9, 36.4)
Males	6,899	39.3 (36.9, 41.6)
Females	6,138	27.3 (24.0, 30.8)
Has item with cigarette brand logo	18,428	40.8 (37.5, 45.2)
Males	9,440	43.7 (41.8, 45.7)
Females	8,988	37.7 (35.9, 40.2)
Seen tobacco adverts on the internet in past 30 days	18,588	45.5 (41.9, 48.8)
Males	9,536	48.4 (45.4, 51.4)
Females	9,049	41.2 (38.6, 44.7)

**Table 2:** Attitudes towards tobacco smoking distributed by gender in Thailand

Characteristic	Number of participants/Total for category	% of total and 95% CI
Felt that boys who smoke have more friends	12,408	58.3 (55.8, 60.8)
Males	6,510	60.7 (58.2, 63.2)
Females	5,898	56.2 (52.2, 60.2)
Felt like girls who smoke had more friends	14,031	20.0 (19.4, 22.8)
Males	7,336	22.5 (20.9, 24.2)
Females	6,695	19.7 (17.1, 22.7)
Felt that boys who smoke are more attractive	14,720	16.5 (15.3, 17.8)
Males	7,310	21.4 (19.3, 23.7)
Females	7,410	12.5 (11.4, 13.7)
Felt that girls who smoke are more attractive	15,411	7.8 (6.7, 8.9)
Males	7,710	10.7 (9.6, 12.0)
Females	7,701	5.3 (3.8, 7.3)
Felt that smoking is harmful to health	18,871	86.7 (85.6, 88.1)
Males	9,695	82.3 (80.2, 84.2)
Females	9,176	90.8 (89.1, 92.3)

### Factors associated with current cigarette smoking

Table 3 reports the results of both bivariate and multivariable logistic regression analysis. In bivariate analysis, the table indicates that older students were less likely to report current smoking than those who were 11–12 years old (OR = 0.42; 95% CI [0.32, 0.56] for 13 years, OR = 0.63; 95% CI [0.44, 0.90] for 14 years olds, and OR = 0.54; 95% CI [0.38, 0.77] for 15 years olds). Males were more than five times likely to report smoking than females (OR = 5.17; 95% CI [4.08, 6.54]). Study participants with one or both parents smokers were 1.98 times likely to report smoking compared to those whose parents were non smokers (OR = 1.98; 95% CI [1.56, 2.53]). Adolescents with most or all friends who smoked were more than twenty times likely to report smoking compared to those who had non smoking friends (OR = 27.45; 95% CI [18.40, 40.96]). Participants who perceived that smoking was harmful were less likely to report smoking (OR = 0.34; 0.25, 0.47]).

**Table 3:** Factors associated with current smoking in Thailand in bivariate and multivariate logistic regression analysis

Characteristic	Current smoking (%, n)	Unadjusted Odds ratio (95% CI^a^)	Adjusted OR (95% CI)
Age (years)			
11–12	20.0 (2144)	1.00	1.00
13	9.6 (4154)	**0.42 [0.32, 0.56]**	**0.49 [0.36, 0.66]**
14	13.6 (5226)	**0.63 [0.44, 0.90]**	**0.56 [0.40, 0.79]**
15	12.0 (3806)	**0.54 [0.38, 0.77]**	**0.59 [0.41, 0.86]**
16–17	15.8 (1214)	0.75 [0.50, 1.13]	0.75 [0.47, 1.20]
Gender			
Female	5.2 (9089)	1.00	1.00
Male	22.0 (9279)	**5.17 [4.08, 6.54]**	**3.64 [2.72, 4.86]**
Parental smoking status			
None	9.6 (9648)	1.00	1.00
One or both parents smokers	17.3 (8805)	**1.98 [1.56, 2.53]**	**1.62 [1.25, 2.11]**
Best friends smokers			
None	4.0 (9445)	1.00	1.00
Some	15.7 (7664)	**4.44 [3.12, 6.32]**	**5.07 [3.54, 7.25]**
Most or all	53.5 (1627)	**27.45 [18.40, 40.96]**	**26.71 [18.26, 39.06]**
Perception that smoking is harmful			
No	27.7 (2362)	1.00	1.00
Yes	11.5 (16309)	**0.34 [0.25, 0.47]**	**0.47 [0.33, 0.66]**

^a^CI = Confidence Interval

In the multivariate analysis, the findings were not different from the bivariate analysis. Current smoking was negatively associated with increasing age and positively associated with male gender, and having friends and/or parents who smoked. The perception that smoking is harmful was negatively associated with smoking in multivariate analysis.

Table 2 indicates that participants were exposed to tobacco adverts through television (35.1%) and internet (45.5%). Four in ten respondents (40.8%) reported having an item with cigarette brand on it. Compared to females, males had higher rates of those who reported having an item with cigarettes logo (p < 0.001) and those who were exposed to adverts on the internet and television (p < 0.001).

Table 3 indicates that most respondents (86.7%) felt that smoking is harmful. 58.3% thought that male smokers had more friends while 20.0% thought so for females. There were twice as many respondents who thought that male smokers were attractive compared to those who thought so for females (16.5% and 7.8%).

## Discussion

The current study reports results from a secondary analysis of the Global Youth Tobacco Survey-2005 for Thailand. Among the studied sample, 22% of males and 5.2% females reported being current cigarette smokers. Many adolescents had been exposed to tobacco advertisements and many perceived smokers favourably (See Tables 1 and 2).

Males were at least 5 times more likely to be current smokers compared to females. This finding is similar to what Rudatsikira et al [26] reported for the Mongolia GYTS. In that study, current cigarette smoking was 5.4 percent among males versus 4.4 percent among females. In logistic regression analysis the following results were obtained: older adolescents were less likely to be current smokers when compared to 11 to 12 year olds; having one or both parents being smokers or having friends who were smokers was associated with being a smoker in the study participants; and adolescents who believed that smoking was harmful to one's health were less likely to be smokers.

It is of interest that younger adolescents reported being current smokers compared to their older colleagues. However the significance of such finding deserves further study. One explanation is that younger adolescents are more likely to experiment and as the adolescent grows older, he or she is less likely to smoke. An alternative explanation is that older adolescents may be smoking equally or more than younger adolescents, but they are less likely to report. This could be solved by collecting biomarkers in future GYTS rounds in order to validate these self-reports on smoking.

Our findings also showed that males were more than 5 times likely to be smokers than females. This is an almost constant finding in GYTS reports and other studies where males are in general, more likely to report being smokers than females [27-30]. In many studies that have reported harmful health behaviours such alcohol misuse, illicit drug use, truancy and violence, males far outnumber females. In case of substance use males report greater use than females for just about every measure [27,28]. There are some exceptions though where the gender disparity does not exist. Ng et al [31], reported that smoking was perceived as evidence of masculinity among Indonesians. Based on the social ecological model we had hypothesized that age, normative beliefs about smoking, sex, parental and peer smoking would be associated with smoking. In both bivariate and multivariable analysis, adolescents who perceived that smoking was harmful to health were in fact less likely to be smokers. This probably suggests that adolescent education on the dangers of smoking may result in prevent them from taking up smoking. Of course, a much less likely reason for the finding would be that current smokers were more likely to downplay the hazards of smoking.

Having friends who are smokers was associated with being a current smoker among the study participants. Due to the cross sectional nature of the data collection in the GYTS, it is not possible to ascribe causation or the sequence of events in an adolescents' smoking trajectory. It is plausible to consider that adolescents who befriend smokers are more likely to be influenced into smoking. It is equally plausible that smoking adolescents are more likely to choose other smokers as their friends [32,33].

Exposure to pro-tobacco advertisements has been reported to be associated with smoking among adolescents in diverse settings. As shown in Table 2, about a quarter of both males and females reported having seen a tobacco brand name on television in the past 30 days, and nearly 50% reported having seen tobacco advertisements on the internet. About 40% reported owning an item with a cigarette brand logo.

Over half of the study participants felt that boys who smoke had more friends than non-smokers while just about one-fifth thought that female smokers had more friends. Similarly, a higher proportion of the study participants reported that boys who smoke were more attractive (16.5%) while a smaller proportion (7.8%) felt that girls who smoke are more attractive. The majority of the study participants felt that smoking was harmful to one's health.

Thailand ratified the WHO Framework Convention on Tobacco Control (WHO FCTC) on November 8, 2004. Thailand has implemented tobacco price-based measures to control tobacco use. The retail price of domestic cigarette brands includes excise and value-added taxes and an additional import tariff is imposed on imported cigarette brands [6]. In 1994, the country increased the level of excise tax [6] and in the subsequent decade this tax policy is credited to have resulted in raising US$1 billion as well as helping to reduce the cigarette smoking prevalence in virtually all age groups.

The Tobacco Products Control Law also prohibited tobacco companies from advertising through sponsorship of events [5]. However, as can be seen in table 2, about a third of the study participants had seen a tobacco advertisement on TV and about 40% own an item with a tobacco brand logo. This observation may imply that enforcement of the law regarding tobacco control is less than being fully effective.

### Limitations of this study

The limitations of the Global Youth Tobacco Survey methodology have been described elsewhere [21,29,30,34]. In brief however, the following are known major limitations. Firstly data are only collected from school-going adolescents. To the extent that a significant proportion of the adolescent population is not in school, the estimates obtained from using these data may be biased. Also, data are only collected from the eligible students that are present in school on the day that the survey is administered in a particular school. No follow-up is made in respect of students who may be absent. However, in a majority of cases, the number of absent, but eligible students is minimal. However as some of the absent students may in fact be truant, and truant students may have different smoking practices than their non-truant peers, the estimates obtained from the GYTS may be slightly be biased. Furthermore, data are collected through self-reporting by the study participants.

There may be intentional as well as unintentional misreporting on any of the questions. However this is minimized by the fact that the survey is self-administered (as compared to interviewer administered) and study participants complete the surveys anonymously. The GYTS does not verify current cigarette smoking status through collection of biological specimens and assessing tobacco by-products such as cotinine and exhaled carbon monoxide [35-38]. However, the fact that the GYTS uses a standard methodology of data collection makes comparisons in prevalence and other estimates among different settings possible.

We can also not claim to have controlled for all important confounders or described all important determinants. A recent study by Primack [39] has shown that exposure to films and music are associated with smoking, but only the relationship between music exposure and smoking persisted after rigorous covariate control. Exposure to books was however associated with lower odds of smoking.

## Conclusion

We have estimated the prevalence of current cigarette smoking among in-school adolescents in Thailand based on data from the Thai Global Youth Tobacco Survey 2005. Being male, having smoking friends or parents and perception that smoking is harmful to one's health was associated with being a current cigarette smoker.

## Competing interests

The authors declare that they have no competing interests.

## Authors' contributions

ER conducted the data analysis and participated in drafting of manuscript, ASM participated in the interpretation of the findings and drafting of the manuscript. SS participated in interpretation of findings and drafting of the manuscript, RHM participated in the interpretation of findings and drafting of manuscript. All authors read and approved the final draft of the manuscript.
